# Influence of land-sea breeze on PM$_{2.5}$ prediction in central and southern Taiwan using composite neural network

**DOI:** 10.1038/s41598-023-29845-w

**Published:** 2023-03-07

**Authors:** George William Kibirige, Chiao Cheng Huang, Chao Lin Liu, Meng Chang Chen

**Affiliations:** 1 Institute of Information Science, Academia Sinica, New Taipei, Taiwan; 2 Social Networks and Human Centered Computing Program, Taiwan International Graduate Program, New Taipei, Taiwan; 3 National Chengchi University, New Taipei, Taiwan

**Keywords:** Network models, Natural hazards

## Abstract

PM$_{2.5}$ prediction plays an important role for governments in establishing policies to control the emission of excessive atmospheric pollutants to protect the health of citizens. However, traditional machine learning methods that use data collected from ground-level monitoring stations have reached their limit with poor model generalization and insufficient data. We propose a composite neural network trained with aerosol optical depth (AOD) and weather data collected from satellites, as well as interpolated ocean wind features. We investigate the model outputs of different components of the composite neural network, concluding that the proposed composite neural network architecture yields significant improvements in overall performance compared to each component and the ensemble model benchmarks. The monthly analysis also demonstrates the superiority of the proposed architecture for stations where land-sea breezes frequently occur in the southern and central Taiwan in the months when land-sea breeze dominates the accumulation of PM$_{2.5}$.

## Introduction

Particulate matter (PM) is composed of air pollutants emitted into the atmosphere through human activities, urban development and industrialization. PM with an aerodynamic diameter smaller than or equal to 2.5 micrometers ($\upmu \hbox {m}$) (PM$_{2.5}$) has been associated with cerebrovascular, cardiovascular, and pulmonary diseases^1–5^. In the Global Burden of Diseases study, PM$_{2.5}$ was ranked the sixth leading cause of human death^6^. One measure against PM$_{2.5}$ harm is to predict precise PM$_{2.5}$ concentrations; many governments have established ground monitoring stations to record PM$_{2.5}$ concentration to enact policies to control excessive atmospheric pollutants.

Taiwan’s Environmental Protection Administration (EPA) has divided Taiwan into seven air quality zones according to geographical and meteorological conditions. Of these air quality zones, the middle and southern air quality zones suffer the most serious air pollution. The literature shows that the characteristics of weather and air pollution are widely considered and play important roles in PM$_{2.5}$ prediction^9^. In addition to PM$_{2.5}$ events caused by local emission, poor atmospheric diffusion conditions, and remote transport, PM$_{2.5}$ concentrations in central and southern Taiwan often reach the national warning threshold due to land-sea breezes^7,8,11^. Simulations from the literature have shown that land-sea breeze events occur with a northwest wind onshore formed during the day and east winds offshore at night^8^. However, this land-sea breeze effect is difficult to detect merely by monitoring station data.

The literature shows that the introduction of machine learning (ML) methods such as feedforward neural networks (FNNs)^12,13^, convolutional neural networks (CNNs)^14^, convolutional long short-term memory (ConvLSTM)^10,15^ and random forests^16–18^ improve the performance of PM$_{2.5}$ prediction. Recently, the development of deep neural network (DNN) approaches has overcome the weakness of other ML methods with their ability to capture complex interactions between datasets from different domains^19^. In our case, the introduction of DNN techniques facilitates the learning of spatio-temporal variation and the distribution of air pollutants from massive datasets. The presence of unknown factors also affects PM$_{2.5}$ prediction . For better prediction, the ensemble models (EMs) produce the softmax-weighted average of several ML model outputs to outperform DNNs^20^. AdaBoost (AD)^21^, generalized additive models (GAM)^22,23^, random forests (RF)^21,23^, and extreme gradient boosting (XGBoost)^21,22,24^ are popular EMs for PM$_{2.5}$ prediction. Recently, the composite neural network^25^ has outperformed the EM methods in PM$_{2.5}$ prediction^10,26^. A composite neural network consists of individually pre-trained DNN components, each of which utilizes knowledge from datasets; component outputs are then connected as an acyclic tree. The leaf outputs are weighted by trained variables and collectively taken as an ensemble node, instead of being softmax weighted as in EM.

In this work, we build a remotely transported pollutants (RTP) model^10^, a composite neural network consisting of two DNN components pre-trained by heterogeneous datasets from multiple sources to improve PM$_{2.5}$ prediction in southern and central Taiwan. We not only train the PM$_{2.5}$ prediction model using local meteorological and air pollution monitoring data, but we also introduce large-scale satellite images of East Asia to aid our model in capturing the spatiotemporal distribution of remotely transported PM$_{2.5}$. To capture the land-sea breezes that play an important role in PM$_{2.5}$ prediction in southern and central Taiwan, large-coverage wind features are also introduced. According to the observation in “Grouping of monitoring stations” and “Land-sea breeze”, the proposed model should yield better PM$_{2.5}$ prediction results at stations where land-sea breeze frequently occur in months when land-sea breeze dominates PM$_{2.5}$.

## Materials

### Study region and air quality data

The study region is located in the south and central part of Taiwan between latitude $21^\circ 25^\prime$ and $24^\circ 15^\prime$ north and longitude $120^\circ 12^\prime$ and $120^\circ 58^\prime$ east as shown in Supplementary Fig. S1. We created a grid area of 234$\times$80 = 18720 km$^2$ that covers the study area for the subsequent data preprocessing. Each individual grid cell has a spatial resolution of 1 km.

The EPA monitoring stations detect air pollutants concentration values such as PM$_{10}$ with a diameter of 10 $\upmu \hbox {m}$, nitrogen dioxide (NO$_2$), other nitrogen oxides (NOx), ozone (O_3_), carbon monoxide (CO) and sulfur dioxide (SO2). All of which strongly influence the formation and future status of PM$_{2.5}$. In this work, we collected hourly detected air pollutant data for 3 years (2014, 2015, 2016) from the Taiwan EPA (https://opendata.epa.gov.tw) as model input.

### Aerosol optical depth data from MAIAC algorithm

Aerosol optical depth (AOD) products are typically generated by dark target (DT) and deep blue (DB) algorithms at spatial resolutions of 3 to 10 km. However, AOD retrieval is challenging, especially when thick smoke is observed by satellite-based monitoring devices, which view the smoke as clouds. This makes the retrieved AOD data unreliable.

Multiangle atmospheric correlation implementation (MAIAC) is an advanced AOD retrieval algorithm based on time series analysis that has been proven reliable for predicting PM$_{2.5}$^27^. The accuracy of MAIAC AOD in China and East Asia has been validated by the AErosol RObotic NETwork (AERONET) ground measurement network^28^. Given MAIAC’s strong performance and global coverage, we use these data to capture information on remote PM$_{2.5}$ transported long distances, for example, from one country to another^10^.

In this work, we collected 3 years (2014, 2015, and 2016) of MAIAC AOD data at a 1$\times$1 km$^2$ spatial resolution from NASA.(https://ladsweb.modaps.eosdis.nasa.gov) The AOD products cover two tiles from the investigation area (h28v06 and h29v06). The coordinates of the four corner points for h28v06 are ($19^\circ 56^\prime$N, $106^\circ 3^\prime$E), ($30^\circ 3^\prime$N, $115^\circ 5^\prime$E), ($29^\circ 59^\prime$N, $127^\circ 2^\prime$E) and ($19^\circ 53^\prime$N, $117^\circ 2^\prime$E). The coordinates of four corner points for h29v06 are ($19^\circ 56^\prime$N, $116^\circ 41^\prime$E), ($30^\circ 3^\prime$N, $126^\circ 37^\prime$E), ($29^\circ 59^\prime$N, $138^\circ 34^\prime$E) and ($19^\circ 52^\prime$N, $127^\circ 41^\prime$E). AOD preprocessing is described in “Data preprocessing”.

### Remote meteorological data

PM$_{2.5}$ can float in the air for 4 to 7 days^29^ and can be transported from one place to another with the help of meteorological features. Meteorological features are also involved in the formation of PM$_{2.5}$^29^.

We used 3 years (2014, 2015, and 2016) of meteorological data from two different sources available in the remote area to capture more remote pollutants. The first source is data on temperature, pressure, vertical velocity (VVEL), absolute vorticity (ABSV), lifted index (LFTX), wind speed (ws) and wind direction ($\theta$) at pressure levels from 10 mb (millibars) to 1000 mb (total 148 features) from the National Center for Environmental Prediction Final (NCEP FNL) Operational Global Analysis data.(https://rda.ucar.edu/datasets/) NCEP FNL data is provided in 2$\times$7 grids which covers $27^\circ \hbox {N}$ to $29^\circ \hbox {N}$ in latitude and $120^\circ \hbox {E}$ to $127^\circ \hbox {E}$ in longitude at six-hour intervals. We pre-processed the data and converted them to hourly intervals, as explained in “Data preprocessing”.

The second source is buoy monitoring stations that record the hourly wind speed and direction over the oceans. The ocean wind (OW) influences the diurnal variation of PM$_{2.5}$ in central and southern Taiwan^11^. Therefore, we create a grid area that covers Each grid cell has a spatial coverage of 1$\times$1 km$^2$. We constructed wind direction and wind speed feature maps by filling non-observed grid cells using kriging interpolation based on wind direction and wind speed features Center Weather Bureau (CWB) stations and buoy weather monitoring devices. Another preprocessing is described in “Data preprocessing”.

By interpolating non-observed grid cells with CWB stations on land and buoy weather monitoring devices on the ocean, we assemble wind feature maps that are reliable within our research area, which is encircled by buoy monitoring devices.

### Local meteorological data

The dispersion and transportation of PM$_{2.5}$ is strongly influenced by meteorological features (rainfall, pressure, temperature, humidity, wind speed, and wind direction)^27^. In this work, we downloaded these features from the CWB website,(http://opendata.cwb.gov.tw/index) which included hourly weather and weather forecast data from 337 monitoring stations. We preprocessed the data as explained in “Data preprocessing” using spatial interpolation to populate all non-observed grid cells and vectorize the wind speed and wind direction data as described in “Wind feature vectorization”.

## Methods

### Wind feature vectorization

Our wind feature maps derive from wind features composed of speed and direction. Wind direction data are usually represented in polar coordinates, which must be converted to vector form. We vectorized the wind feature from wind speed at a particular angle into meridional (v-wind) and zonal (u-wind) components. To isolate the wind speed feature from the direction features, we then normalized the u-wind and v-wind by dividing them by the wind speed to yield the meridional and zonal components of the unit wind direction vector.

### Data preprocessing

Data preprocessing includes conversion from monitoring station-based areas to a grid, linear interpolation, spatial interpolation to populate empty grid cells, data cleaning, and spatial downscaling.

For AOD, NCEP meteorological data and ocean wind which are input to STRI model in “Modeling methods”, we vectorized the wind direction into zonal and meridian components of the meteorological dataset (NCEP) as described above. We also used linear interpolation to convert the meteorological dataset (NCEP) to hourly intervals from a six-hour interval.

We cleaned the MAIAC AOD data at 550 nm by filtering out poor quality grid values, after which we interpolated using the remaining grid cells. We also downscaled the spatial dimension of each remote tile (h28v06 and h29v06) to 300$\times$300 km$^2$ from 1200$\times$1200 km$^2$ using maximum pooling^15^ to fit the available memory of the GPU. Then, we repeated the daily reading of each grid cell 24 times to match the hourly interval of other datasets.

**Figure 1:**
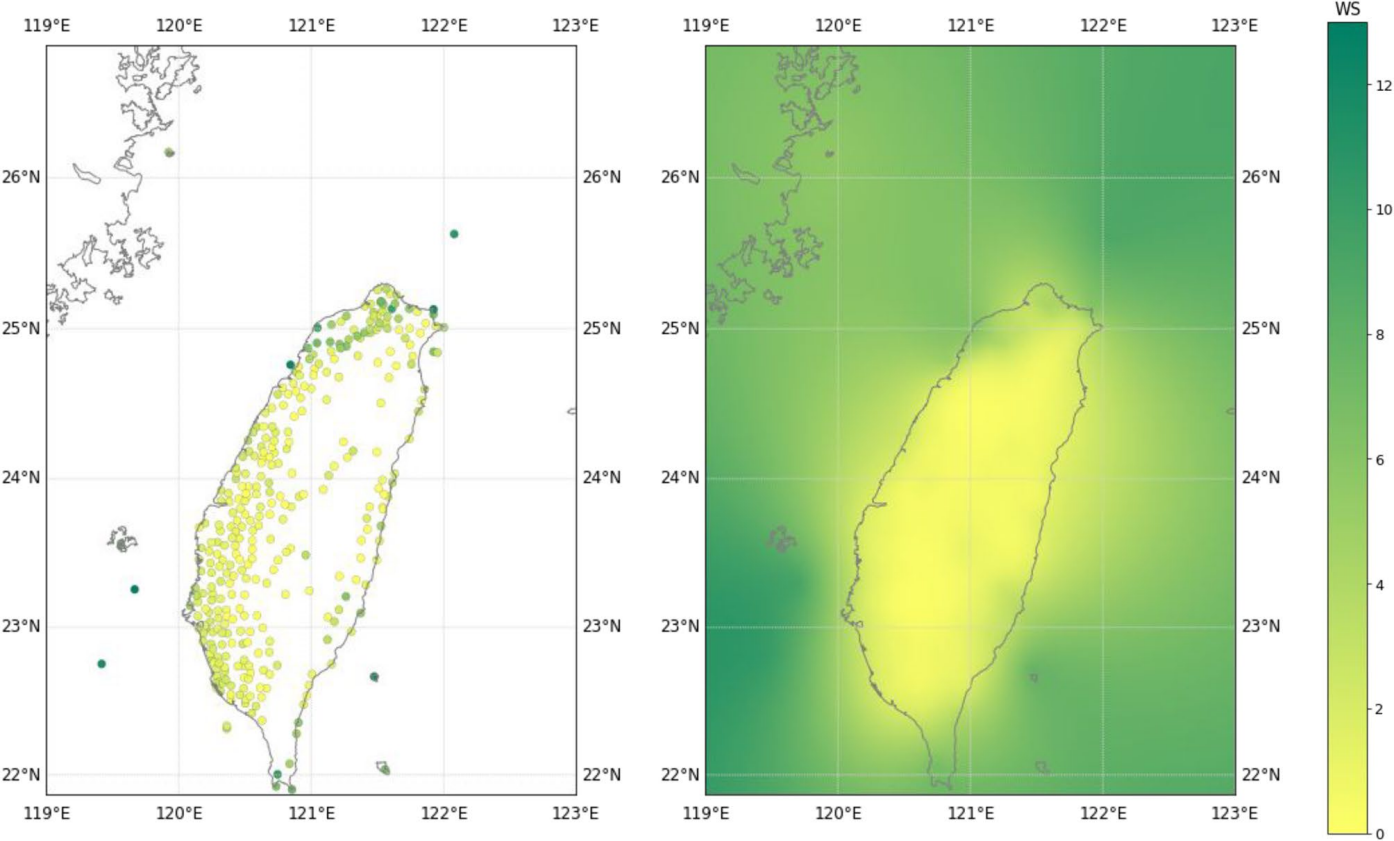
Left side: CWB and buoy monitoring stations. Right side: distribution of ocean wind dataset after kriging interpolation.

To capture the spatio-temporal characteristics of the speed and direction of the ocean wind over the sea, we created a grid area (492$\times$396 = 194,832 km$^2$) inside the remote area with each grid cell covering 1$\times$1 km$^2$. We created a feature map by populating the dataset in the grid area according to the latitude and longitude coordinates of the monitoring stations (CWB and buoys). We used kriging interpolation to populate the remaining grid cells that did not match the station coordinates. Shown in Fig. 1 is an example of the results after kriging interpolation on the CWB and buoy dataset. Maximum pooling was applied to the kriging interpolated feature map to reduce the spatial dimensions to 246$\times$198 km$^2$ to match the memory of the computing resource.

For air quality, weather, and weather forecast feature maps that are input to the base model in “Modeling methods”, we converted the study regions to the grid area (234$\times$80 cells) and created the feature map by populating the grid cells with the observed air quality and meteorological data according to the coordinates of the monitoring stations (37 EPA, 174 CWB) and using four nearest neighbors (4-NN) to populate grid cells outside these coordinates.

### Modeling methods

The proposed composite neural network models—RTP with DNN components (base, STRI)—were trained over 2 years (2014, 2015) of data and tested on one year (2016). All models were constructed using Keras with a TensorFlow backend and trained on an NVIDIA GPU with 11 GB of memory.

#### STRI component

The spatiotemporal remote information neural network (STRI)^10^ is a component of the RTP model that captures remotely transported PM$_{2.5}$ and predicts local PM$_{2.5}$ concentration. We added ML layers (CNN, ConvLSTM) to the STRI model to capture the spatiotemporal characteristics of the new heterogeneous dataset (AOD, meteorology, ocean wind). We included the AOD data to provide more spatial-temporal information on PM$_{2.5}$ remotely transported towards Taiwan.

**Figure 2:**
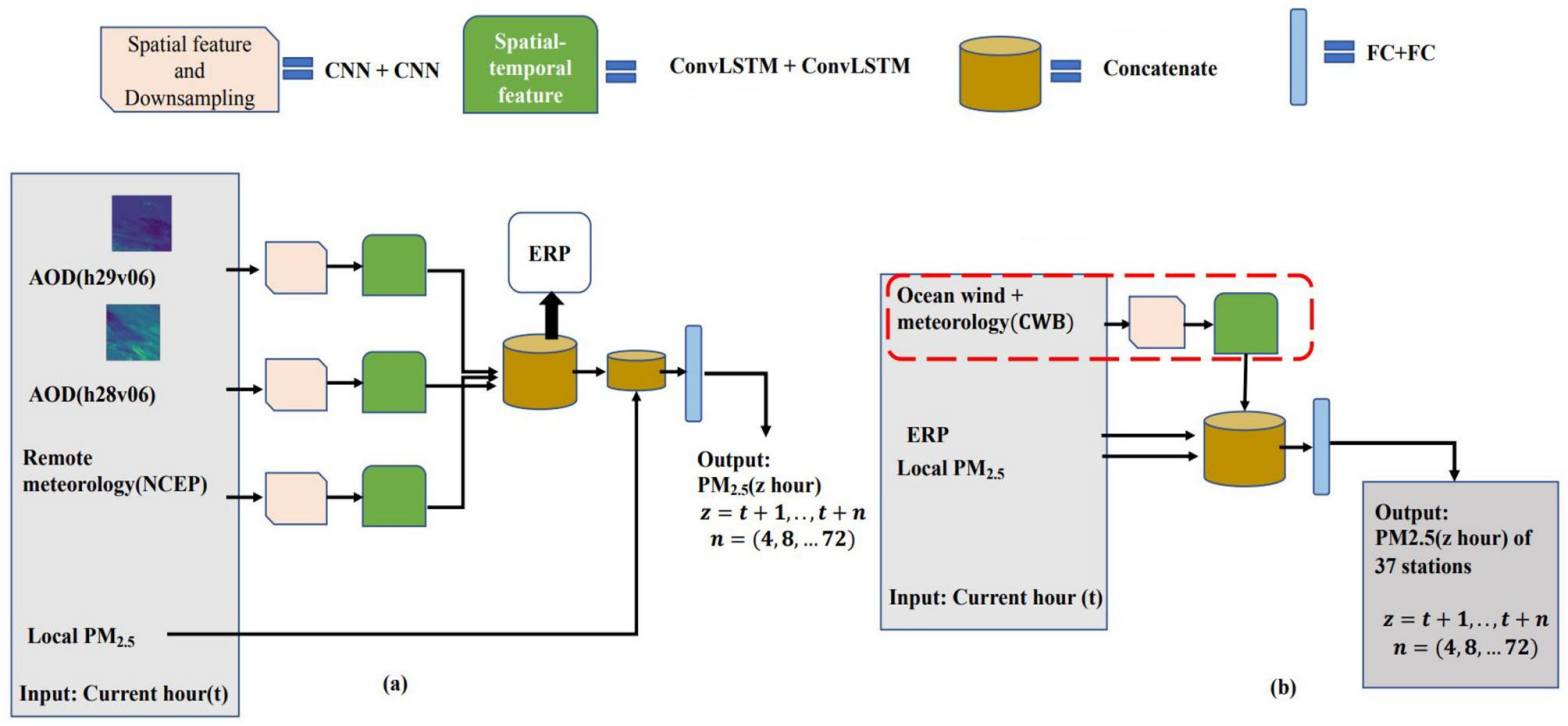
STRI model components STRI_fe (**a**) and STRI_p (**b**) with modifications indicated by red dashed line.

In this work, the large STRI model with multiple layers of ML predicts the local PM$_{2.5}$ concentration of 37 EPA stations . The model uses large and heterogeneous datasets (AOD, meteorology, ocean wind) with local PM$_{2.5}$ as input. In Fig. 2, STRI_fe inputs 300$\times$300 sized AOD satellite image, 2$\times$7$\times$148 sized NCEP meteorological grid data and the local PM$_{2.5}$ value; STRI_p inputs Kriging interpolated ocean wind grid data (246$\times$198) and the embedding generated from STRI_fe. The idea is to capture spatiotemporal characteristics of heterogeneous datasets in different spatial scales, concatenate these, and then merge them with local features (PM$_{2.5}$) to predict local PM$_{2.5}$ concentration.

Furthermore, to fit the large STRI model into the GPU memory, we divided the model into two components, as shown in Fig. 2. STRI_fe, the first component^10^, is used for the extraction of remote pollutants (ERP) given the AOD input from two tiles with their meteorology dataset. STRI_p, the second component, is used for the prediction given the ERP input, local features, and spatiotemporal features of ocean wind (Fig. 2). The detailed configuration of STRI model is described in detail in Supplementary Table S1.

After dividing the model into two components, we borrowed techniques from previous work^10^ to fine-tune the individual components with fewer training parameters to improve the final prediction results.

#### Base component

The base model^10^ is a component of the RTP model that predicts PM$_{2.5}$ concentration using local features only. The input to the base model is the air quality feature maps interpolated from EPA monitoring stations , the weather and weather forecast feature maps interpolated from CWB monitoring stations that covers the study area, and the prediction hour value to predict PM$_{2.5}$ of 37 EPA stations. . The model is described in detail in Supplementary Fig. S2.

#### RTP model

Given the prediction output of its pre-trained components (STRI and the base model), the RTP model outputs the final PM$_{2.5}$ predictions for the 37 EPA stations by hour. The RTP model is described in detail in Supplementary Fig. S2.

## Evaluation

### Metrics

We evaluated the proposed models using the root mean square error (RMSE), which measures the difference between the predicted PM$_{2.5}$ and its true value. In this work, the RMSE is the squared mean of the error between the ground truth and the predicted value at every hour among the monitoring stations of interest:

1 $$\begin{aligned} RMSE = \sqrt{\frac{1}{n}{\sum }_{t=1}^{T}{\sum }_{i=1}^{n}{(y_{t,i} -{\hat{y}}_{t,i})^2}}, \end{aligned}$$

where $y_{t,i}$ and $\hat{y_{t,i}}$ are the true and predicted value of monitoring station *i* at hour *t* respectively, *T* is the length of the prediction sequence and *n* is the total number of monitoring stations.

### Evaluation of proposed architecture

We conducted experiments to show the the model performance for the next 3 days (72 h) PM$_{2.5}$ prediction at 4-hour intervals by comparing them with benchmarks and also to evaluate the contribution of each input feature to the prediction performance. In our architecture, each model was trained with the corresponding data from 2014 to 2015 and evaluated with the data from 2016.

1. We first compared the prediction performance of RTP_ow with its components (STRI pre-trained with the ocean wind and the base model) to evaluate the improvement of the composite neural network architecture with respect to PM$_{2.5}$ prediction.
2. After comparing the PM$_{2.5}$ prediction performance of the RTP model with its components, we compared the RTP model with other ensemble models (ADA, GAM, RF, XGB). RTP and the ensemble models use the same inputs: the prediction output of STRI and the base model. The main objective of these comparisons is to show that RTP outperforms its pre-trained components and other ensemble techniques.
3. Furthermore, We compared the PM$_{2.5}$ prediction performance of RTP models composed of STRI pre-trained with ocean wind (RTP_ow) and RTP models composed of the STRI pre-trained without ocean wind (RTP_no_ow) components to evaluate the effect of pre-training with ocean wind data on PM$_{2.5}$ prediction. Before this experiment, we grouped the 37 EPA stations of interests into two groups by ranking the frequency of land-sea breeze occurrences (The detailed grouping method is described in “Grouping of monitoring stations”). In this comparison, we averaged the RMSE of each group of stations during prediction hours to investigate the effect of ocean wind data on PM$_{2.5}$ prediction performance at stations where land-sea breeze frequently occur.
4. Finally, we present the monthly PM$_{2.5}$ prediction performance of models trained under the proposed architecture by averaging the RMSE of the 37 stations at each prediction hour in each month to compare the model performance during months when land-sea breezes affect southern and central Taiwan to the performance during the rest of the year. In addition to the RMSE of the 37 stations, we also averaged the RMSE of each group of stations grouped in “Grouping of monitoring stations” at each prediction hour to investigate the effect of ocean wind data on PM$_{2.5}$ prediction performance at stations where land-sea breeze frequently occur in each month.

### Grouping of monitoring stations

To evaluate whether the introduce of ocean wind data improves the PM$_{2.5}$ prediction performance at stations where land-sea breeze frequently occur, we selected the 28 stations land-sea breeze frequently occur and annotated these as LS stations, as listed in Supplementary Table S2. The processes we determine the LS sites are listed below. First, calculate the average daily wind direction (from 7am to 6pm) and the night wind direction of each site within 2014 to 2016; Second, calculate the counts of days when the daytime average wind direction lies between $157.5^\circ$ and $337.5^\circ$ (this step aims at counting the days when the daytime wind comes from the sea), and the difference of average wind direction in the daytime and nighttime is greater than $135^\circ$ (this threshold indicates the significant diurnal wind direction variation); Third, select sites of top 28 counts as LS sites. The remaining nine stations (Xianxi, Lunbei, Mailiao, Taixi, Xingang, Puzi, Xinying, Annan and Hengchun) are annotated as normal stations.

### Land-sea breeze

Many studies present land-sea breeze with backward trajactory simulation for few hours period. To provide a synoptic observation of land-sea breeze in different season, a quantified metric, Jensen-Shannon divergence (JS divergence), is used to measure land-sea breeze through statistics from monitoring stations’ observation. JS divergence is a method of measuring the similarity between two probability distributions. The lower JS divergence of two distributions is, the closer the two distributions are. As shown in Supplementary Fig. S3, we present the daytime and nighttime wind directions of each month in two discrete probability distributions. In practice, we present each probability as an array of 8 elements (the elements represent the probability of 8 principal wind directions within a month). Then, we calculate the similarity of the two distribution with the following JS divergence formula:

$$\begin{aligned} JSD(P||Q) = \frac{1}{2}\sum _i{P_i\log \frac{P_i}{\frac{P_i+Q_i}{2}}} + \frac{1}{2}\sum _i{Q_i\log \frac{Q_i}{\frac{P_i+Q_i}{2}}} \end{aligned}$$

where *P* and *Q* represent the discrete probability distribution in the 8 principal wind directions in day and night, respectively; *i* represents each principal wind direction.

Thus, JS divergence is able to represent the diurnal wind direction variation in monthly probability.

In Supplementary Fig. S4, we presents four cases (Annan, Mailiao, Nanzi and Linyuan) of stacked bar plot which represent the distribution that PM$_{2.5}$ events of different AQI index level occur under eight principal wind direction every months in 2016. For each case, top row is the daytime distribution and the bottom row is the nighttime. JS divergence value is shown in top diagram. When land-sea breeze dominates, the diurnal wind direction distribution shift is recognizable, and JS divergence value is greater than 0.3.

From the four stations, we found JS divergences are relatively higher from April to August. This indicates that land-sea breeze dominates from April to August. For Nanzi and Linyuan, which are LS stations, the occurrences of PM$_{2.5}$ concentration at *Unhealthy for sensitive groups* and *Unhealthy* AQI index level grow at nighttime from March to May. This indicates that the PM$_{2.5}$ concentration at LS stations is dominated by land-sea breeze from March to May. However, PM$_{2.5}$ concentration is mostly under *Good* and *Moderate* AQI index level in June, July and August. In summer, strong vertical convection also influenced PM$_{2.5}$ and attributes to low PM$_{2.5}$ concentration. Hence, the proposed RTP is expected to perform well especially during March and May.

In January, February, November and December, the distributions between daytime and nighttime look similar and the JS divergences are small. The bars at northeast, north and northwest are relatively high. This indicates northeast monsoon dominates the whole day during the four months. However, for Nanzi and Linyuan stations, JS divergence values are relatively higher than the other two stations during winter. JS divergences of Linyuan station even keep above 0.3 for the whole year. As we have known that Linyuan and Nanzi are selected as the LS stations, this quantified metric clearly demonstrate that the LS stations are prone to be influenced by land-sea breeze.

The observations above help explaining the RTP performance in each month in “Monthly analysis”.

## Results

### RTP and its components

**Figure 3:**
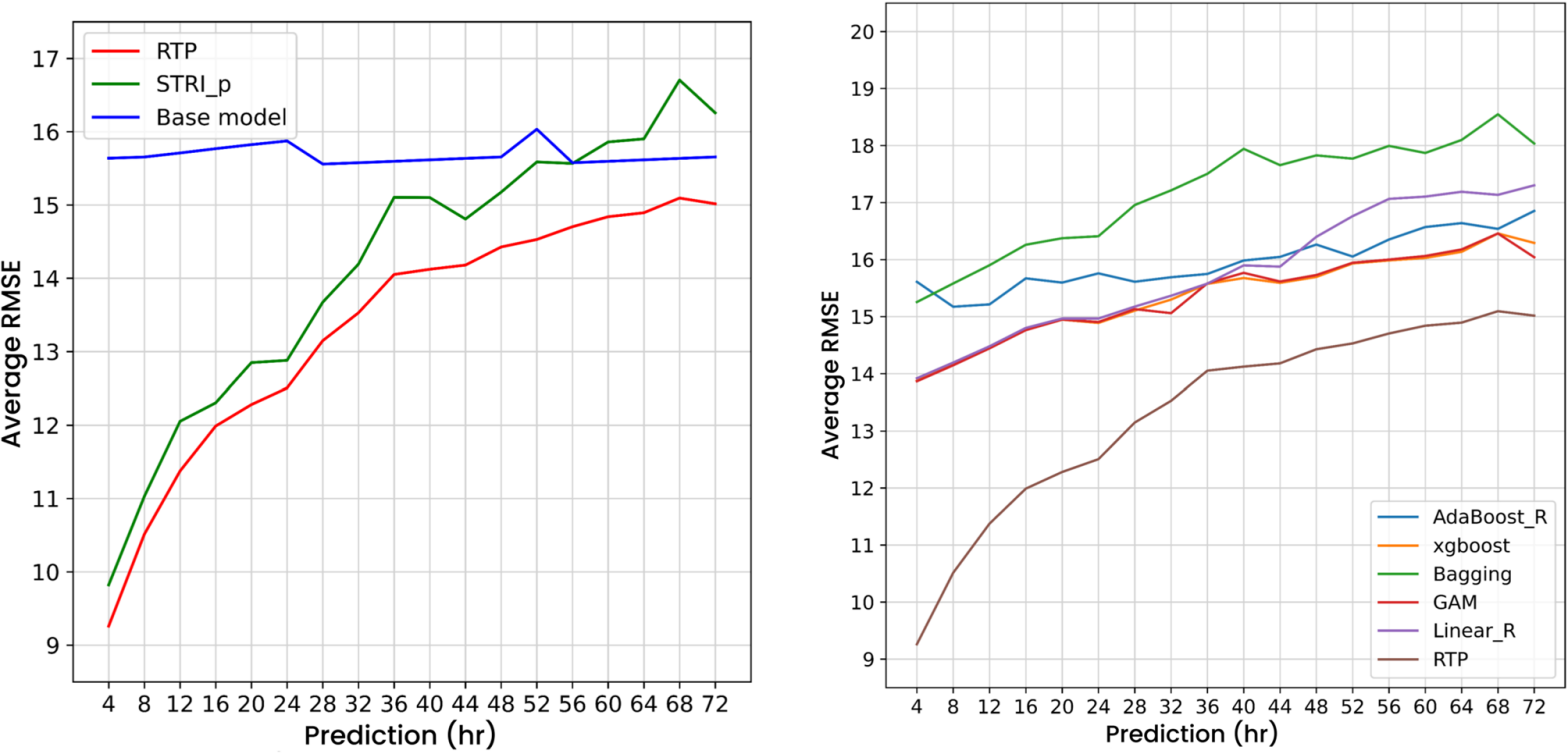
Left: average RMSE for RTP and its components. Right: average RMSE of RTP and other ensemble models.

Figure 3 (left) shows that RTP and STRI both significantly outperform the base model from prediction hour 4 to 32. However, for the prediction hour after 32, STRI is worse than the base model, while RTP exhibits the best PM$_{2.5}$ prediction performance. This experiment shows that composite neural network architecture significantly improves PM$_{2.5}$ prediction compare to its components.

### RTP model and other ensemble models

As ensemble models such as AdaBoost, generalized additive models, random forests, and XGBoost have been widely used for PM$_{2.5}$ prediction, we further compared the RTP model trained under the proposed architecture with these models. In this experiment, we input the prediction output from both the STRI and the base model components into the RTP and the ensemble models. In Fig. 3 (right), the RTP model outperforms the ensemble models (ADA, GAM, RF, XGBoost) at every prediction hour. This shows that the proposed composite neural network architecture has the best overall PM$_{2.5}$ prediction performance in southern and central Taiwan with components pre-trained using large-scale AOD, weather, and ocean wind data.

### Effect of pre-trained components on RTP model

**Figure 4:**
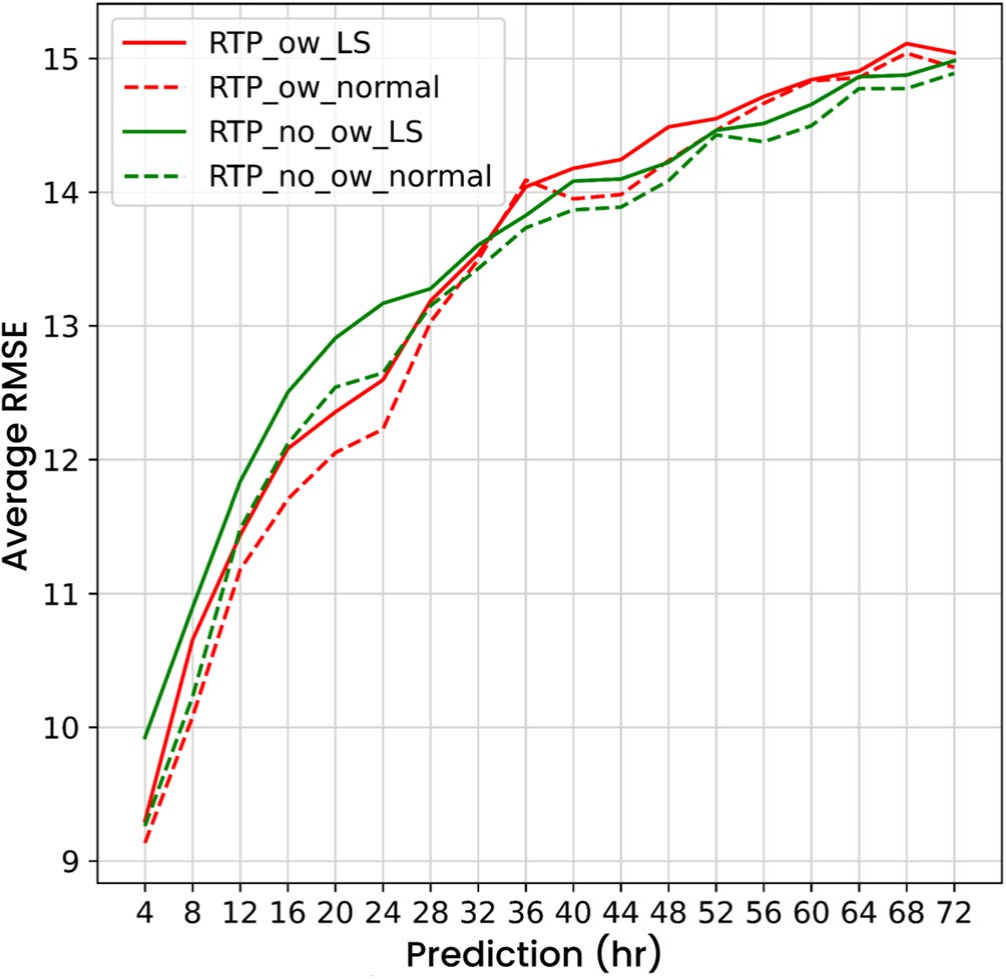
Average RMSE of LS stations (solid line) or normal stations (dashed line) in every prediction hour for RTP pre-trained with (RTP_ow) or without (RTP_no_ow) ocean wind.

To evaluate the effect of ocean wind data on RTP model with respect to PM$_{2.5}$ prediction, we compared RTP composed of two different pre-trained STRI models: STRI pre-trained with PM$_{2.5}$ and AOD data (RTP_no_ow), and STRI pre-trained with PM$_{2.5}$, AOD, and ocean wind data (RTP_ow). In Fig. 4, comparing RTP_ow to RTP_no_ow shows that ocean wind features does help PM$_{2.5}$ prediction performance from prediction hour 4 to 28; however, Table 1 shows that in terms of average RMSE during the prediction hours, RTP_ow outperforms RTP_no_ow. This shows that ocean wind helps pre-trained components of composite neural network models to improve the overall PM$_{2.5}$ prediction performance during 72-hour prediction.

**Table 1:** Average RMSE for land-sea (LS) and normal stations for RTP pre-trained with (RTP_ow) and without (RTP_no_ow) ocean wind.

	RTP_no_ow	RTP_ow
LS stations	13.4834	13.4037
Normal stations	13.2319	13.2190
All stations	13.4222	13.3588

### Monthly analysis

**Figure 5:**
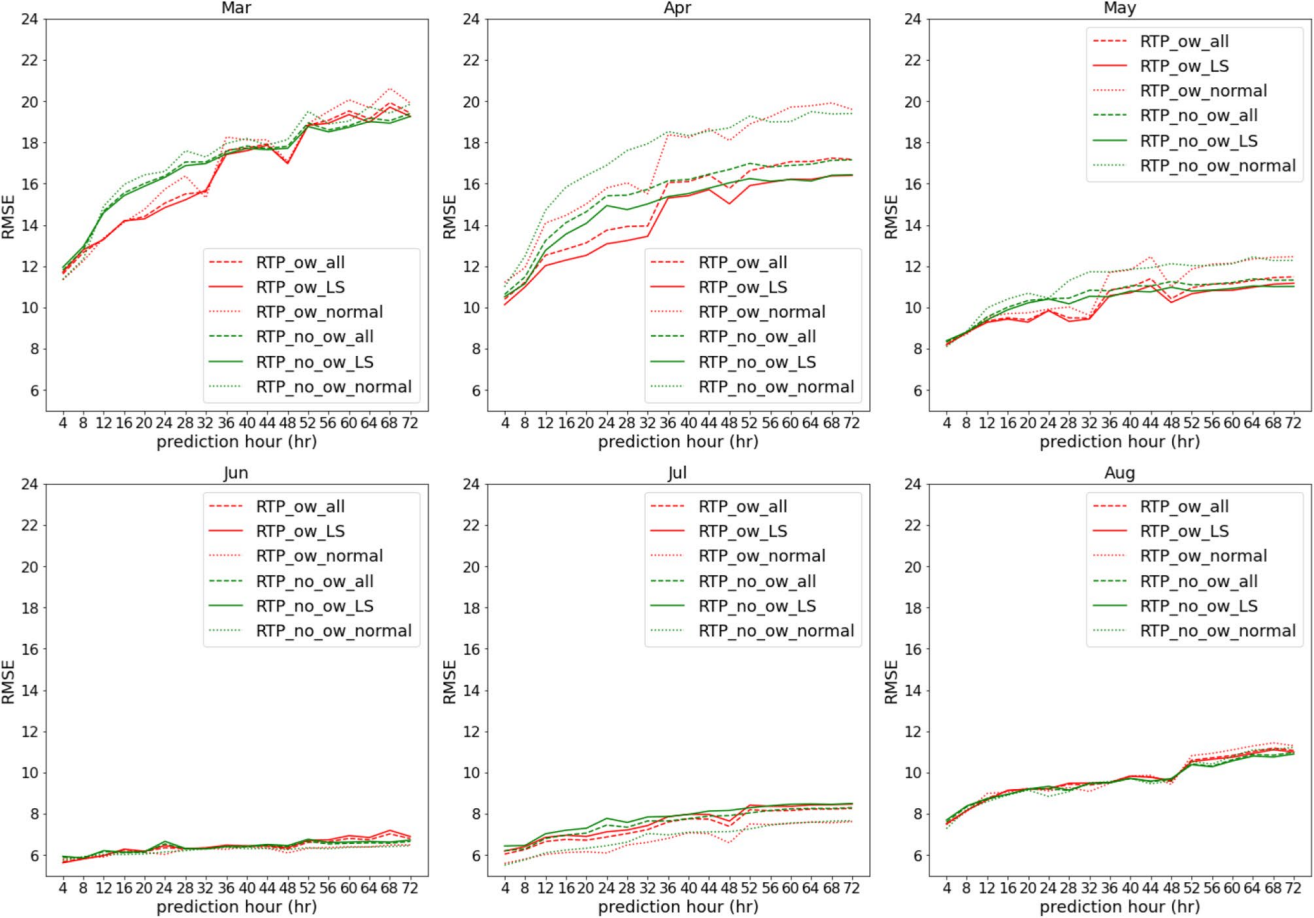
Monthly average in RMSE.

In Fig. 4, although RTP_ow significantly improves the PM$_{2.5}$ prediction performance from prediction hour 4 to 28, RTP_ow_normal is obviously better than RTP_ow_LS through out every prediction hours, which means that the proposed architecture performs worse at LS stations during the whole testing period (2016). However, land-sea breeze does not dominate PM$_{2.5}$ throughout the year according to the observations in “Land-sea breeze”. To evaluate whether the proposed composite neural network architecture that introduces ocean wind yields improved the PM$_{2.5}$ prediction performance for stations where land-sea breeze frequently occur, we separated the PM$_{2.5}$ prediction performance of RTP_ow for all 2016 into intervals of one-month for both LS and normal stations, as shown in Fig. 5. These monthly prediction results show that, in terms of average RMSE, RTP_ow for LS stations outperform RTP_ow for normal stations during prediction hours in March, April,and May. Importantly, RTP_ow for LS stations shows better performance compared to RTP_no_ow for LS stations from prediction hour 4 to 32 in March, April,and May. In June and August, when PM$_{2.5}$ pollution is the lowest in the year, both RTP_ow and RTP_no_ow have similar performance no matter for LS stations or normal stations.

In Supplementary Fig. S3, we present the monthly prediction results for autumn and winter: clearly, RTP_ow exhibits superior prediction performance for normal stations in September, October, December, and January. Although LS stations show higher JS divergence throughout the whole year according to Supplementary Fig. S4, northeast monsoon mainly dominates PM$_{2.5}$ events in *Unhealthy* level, which weaken the effect of land-sea breeze. In summary, the proposed composite neural network architecture that introduces ocean wind data, RTP, produces an improved PM$_{2.5}$ prediction performance for stations where land-sea breeze frequently occur in southern and central Taiwan in months when land-sea breeze dominates PM$_{2.5}$.

## Conclusion

We propose a composite neural network architecture that uses components pre-trained with large-scale weather features and ocean wind to predict PM$_{2.5}$ in southern and central Taiwan. The neural network RTP_ow, which uses STRI, pre-trained with PM$_{2.5}$, AOD, large-scale weather features and ocean wind features as components, achieved the best overall PM$_{2.5}$ prediction performance compared to its individual components and other ensemble models. Monthly analysis reveals that the proposed model yields improved PM$_{2.5}$ prediction for LS stations in southern and central Taiwan in months when land-sea breeze dominates PM$_{2.5}$.

## Supplementary Information

Supplementary Information.

## Data Availability

The data that support the findings of this study are available from the corresponding author upon reasonable request.
