# Supplementary material for: Influence of land-sea breeze on PM2.5 prediction in central and southern Taiwan using composite neural network
Source: Sci Rep. 2023 Mar 7;13:3827. doi: 10.1038/s41598-023-29845-w (PMC9992406; doi:10.1038/s41598-023-29845-w)
Supplement: Supplementary file 1 — Supplementary Information. [file 41598_2023_29845_MOESM1_ESM.pdf]

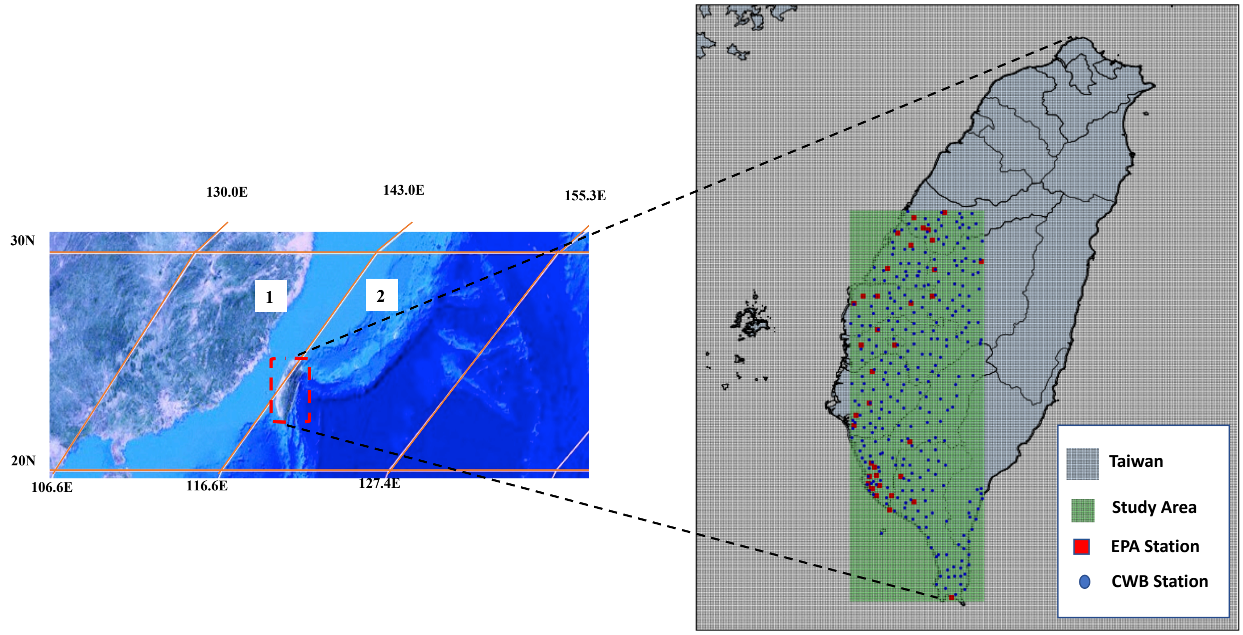

Figure S1. Left side: Two tiles label 1(h28v06), 2(h29v06) with Taiwan in the middle between tiles. Right side: The map of Taiwan after zooming with study area.

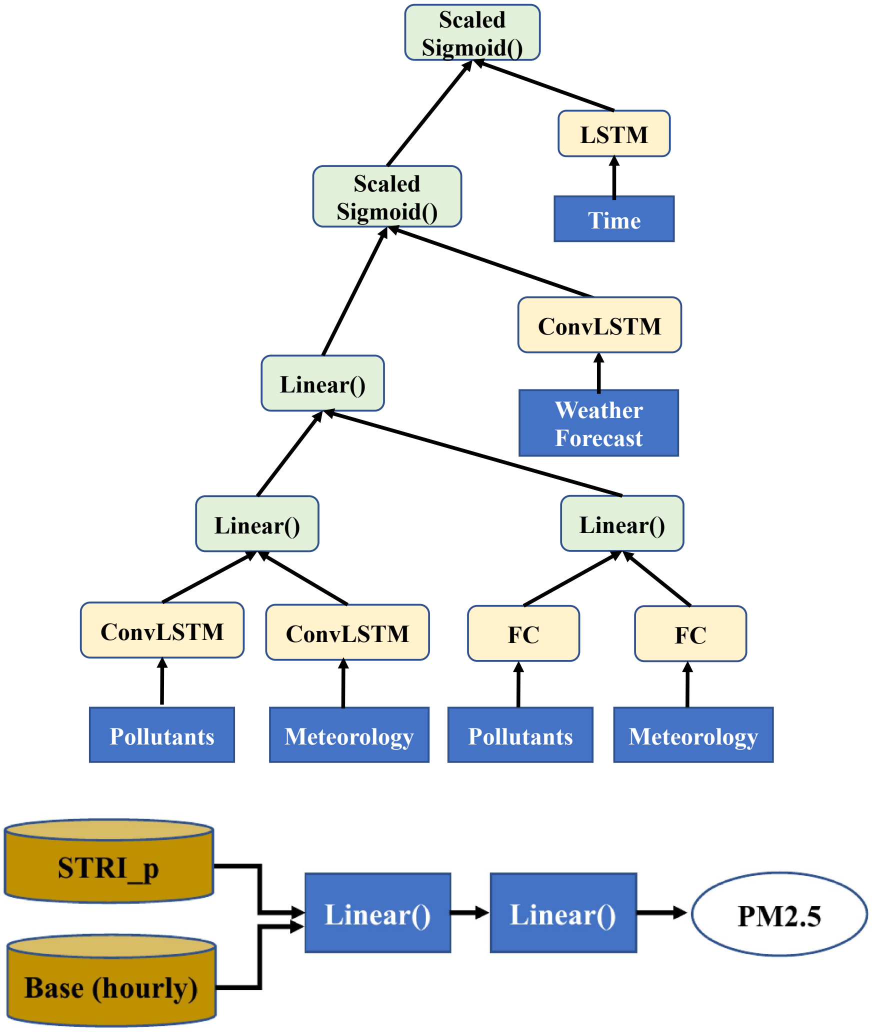

Figure S2. Top: Base model. Bottom: RTP model

(a) DETAIL CONFIGURATION FOR STRI_fe

Tile 1	Tile 2	Remote Meteorological data
**Layer Name**	**Layer Name**	**Layer Name**
3D Average pooling 1x2x2<br>stride 1x2x2<br>padding same	3D Average pooling 1x2x2<br>stride 1x2x2<br>padding same	ConvLSTM 1x3 64 units<br>ReLU
2D CNN 3x3 32 maps ReLU	2D CNN 3x3 32 maps ReLU	BatchNormalization layer
2D CNN 3x3 32 maps ReLU	2D CNN 3x3 32 maps ReLU	ConvLSTM 1x3 32 units<br>ReLU
Dropout rate 0.3	Dropout rate 0.3	Flatten
ConvLSTM2D 3x3 32 units ReLU	ConvLSTM2D 3x3 32 units ReLU	
BatchNormalization layer	BatchNormalization layer	
ConvLSTM2D 3x3 32 units ReLU	ConvLSTM2D 3x3 32 units ReLU	
3D Average pooling 1x10x10<br>stride 1x10x10<br>padding same	3D Average pooling 1x10x10<br>stride 1x10x10<br>padding same	
Flatten	Flatten	
Concatenate		
Repeat vector		
Concatenate: output of this layer is saved as ERP		

(b) DETAIL CONFIGURATION FOR STRI_p

Current Ocean wind + CWB	ERP	Local PM2.5
**Layer Name**	**Layer Name**	**Layer Name**
2D Average pooling 3x3<br>stride 3x3<br>padding same	-	-
2D CNN 5x5<br>stride 4x4 32<br>maps ReLU		
2D CNN 3x3 16 maps ReLU		
2D Average pooling 3x3<br>stride 2x2<br>padding same		
ConvLSTM2D 3x3 32 units ReLU		
BatchNormalization layer		
ConvLSTM2D 3x3 16 units ReLU		
3D Average pooling 1x3x3<br>stride 1x2x2<br>padding same		
Flatten		
Concatenate		
Fully connected 52 Units ReLU		
Dropout rate 0.3		
Fully connected 42 Units ReLU		
Dropout(rate 0.4)		
Fully connected 37 Units linear		

Table S1. STRI model configurations

Table S2. Coastal and inland stations

LS stations	Normal stations
Zhushan	Xianxi
Puli	Lunbei
Chaozhou	Mailiao
Dali	Taixi
Fengyuan	Xingang
Nantou	Puzi
Douliu	Xinying
Linyuan	Annan
Pingtung	Hengchun
Daliao	
Meinong	
Shanhua	
Fengshan	
Nanzi	
Renwu	
Changhua	
Xiaogang	
Qiaotou	
Chiayi	
Zhongming	
Xitun	
Qianzhen	
Fuxing	
Tainan	
Erlin	
Shalu	
Zuoying	
Qianjin	

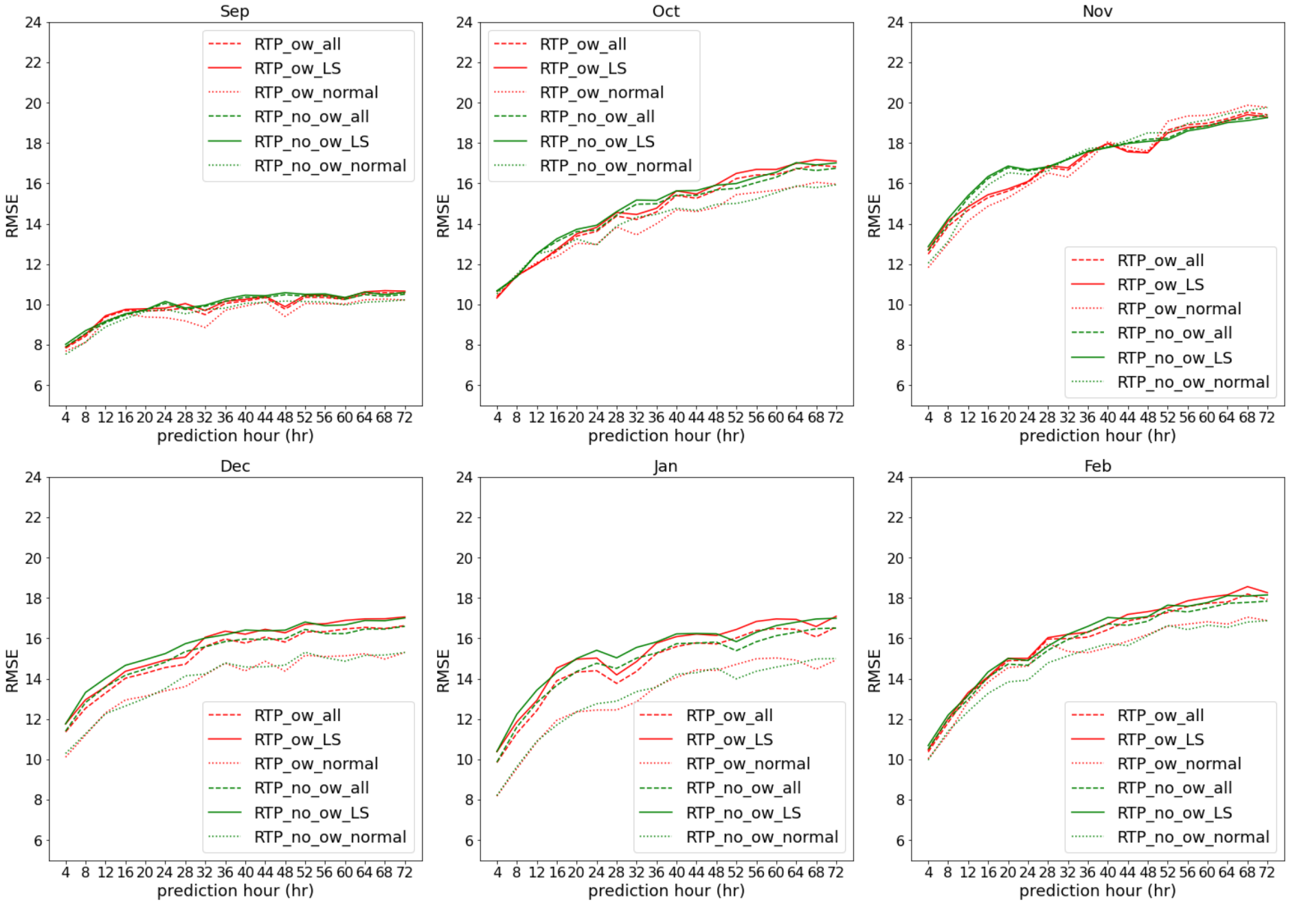

Figure S3. Monthly average RMSE

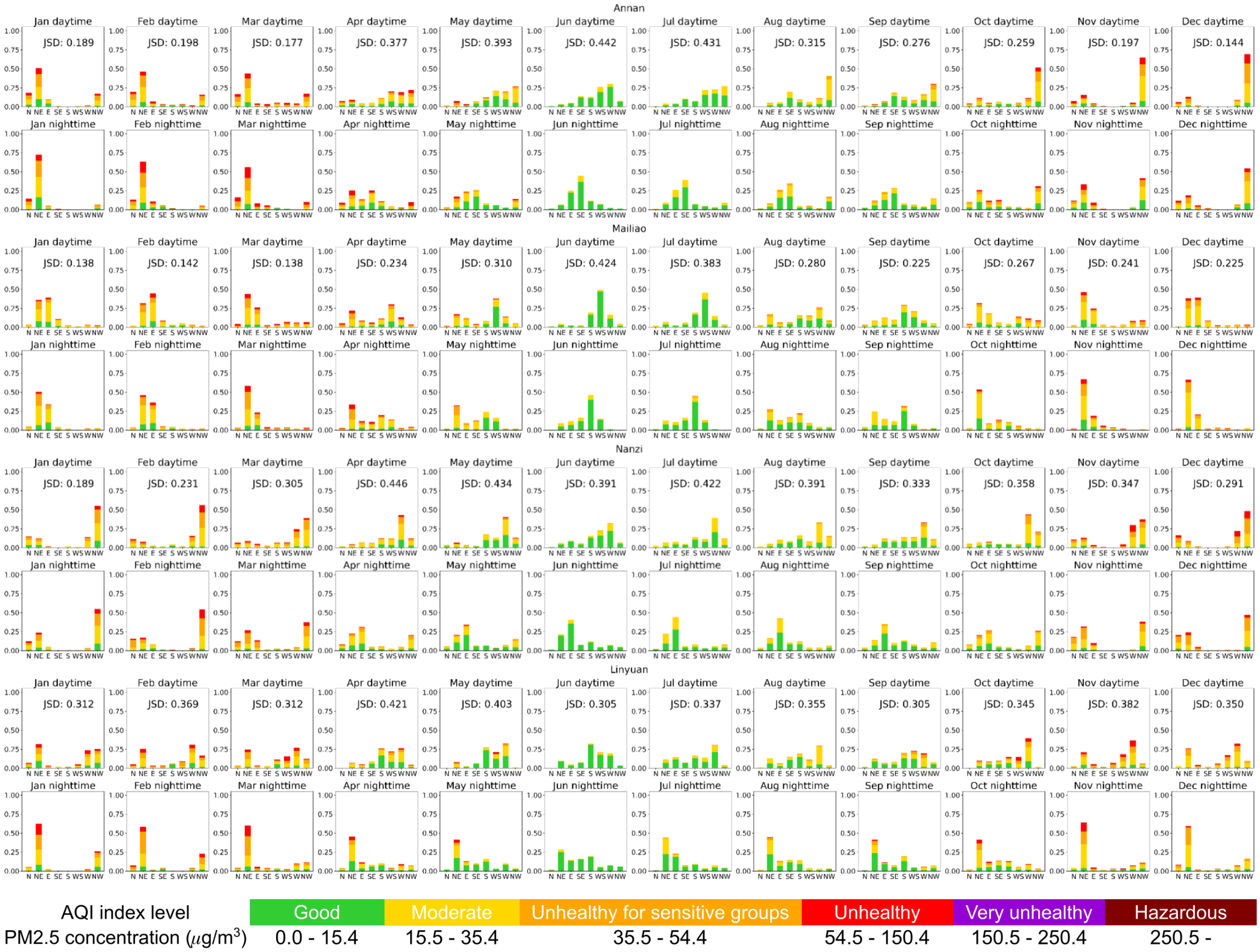

Figure S4. Cases of daytime and nighttime wind direction distribution for each month
